# Genetics of Congenital Heart Disease

**DOI:** 10.2174/157340310791162703

**Published:** 2010-05

**Authors:** Ashleigh A Richards, Vidu Garg

**Affiliations:** 1 Department of Pediatrics, University of Texas Southwestern Medical Center, Dallas, Texas, USA; 2 Department of Pediatrics, The Ohio State University College of Medicine and The Heart Center and Center for Cardiovascular and Pulmonary Research, Nationwide Children’s Hospital, Columbus, Ohio, USA

**Keywords:** Congenital heart disease, genetics, cardiac development.

## Abstract

Cardiovascular malformations are the most common type of birth defect and result in significant mortality worldwide. The etiology for the majority of these anomalies remains unknown but genetic factors are being recognized as playing an increasingly important role. Advances in our molecular understanding of normal heart development have led to the identification of numerous genes necessary for cardiac morphogenesis. This work has aided the discovery of an increasing number of monogenic causes of human cardiovascular malformations. More recently, studies have identified single nucleotide polymorphisms and submicroscopic copy number abnormalities as having a role in the pathogenesis of congenital heart disease. This review discusses these discoveries and summarizes our increasing understanding of the genetic basis of congenital heart disease.

## INTRODUCTION

Congenital heart disease (CHD) is the leading cause of birth defects, and accounts for more deaths in the first year of life than any other condition when infectious etiologies are excluded [1]. With an incidence ranging from 19 to 75 per one thousand live births and present in an even greater proportion of miscarriages, CHD is an important cause of childhood morbidity and mortality worldwide [2]. Despite advances in medical and surgical care, the etiology of CHD is still not completely understood; and with more children with CHD surviving to adulthood and starting families, it becomes even more critical to understand the origins of CHD. Classic studies including the Baltimore-Washington Infant Study have found that CHD is multifactorial, due to both genetic predisposition and environmental influences [3]. Sequencing of the human genome and advances in molecular techniques has led to increasing evidence implicating a stronger role for genetic factors. 

Over the past couple of decades, there has been a greater understanding of the molecular pathways regulating cardiac development. The development of gene targeting technology has led to the generation of a multitude of mouse models with cardiac developmental defects. These studies have led to the identification of numerous transcriptional regulators, signaling molecules and structural genes that are critical for normal cardiac morphogenesis. In addition, multiple genes have been identified that are controlled by these highly conserved molecular pathways. These investigations into the molecular mechanisms of cardiac development have assisted in the identification of genetic etiologies of CHD and provide evidence that many genes may have etiologic roles in human CHD.

In this review, we will discuss the evolution of knowledge regarding genetic causes of CHD, from early evidence that aneuploidy was associated with CHD, to the later use of submicroscopic techniques such as fluorescence-in-situ hybridization, and more recently the identification of single gene mutations as a cause of CHD. We will focus on new developments in the field of cardiac genetics, specifically in relation to the importance of copy number variations and single nucleotide polymorphisms in the development of CHD.

## CARDIAC MALFORMATIONS ASSOCIATED WITH ANEUPLOIDY AND MICRODELETIONS

While most children born with CHD do not have other birth defects, CHD occurs in association with other anomalies or as part of an identified syndrome in 25 to 40% cases [4]. In addition, approximately 30% of children with a chromosomal abnormality will have CHD [5]. Aneuploidy, or abnormal chromosomal number, accounts for a significant proportion of CHD (Table **1**). Fifty percent of individuals born with Trisomy 21 have CHD, ranging from atrial and ventricular septal defects to atrioventricular canal lesions. In Trisomy 13, the incidence increases to 80%, with heterotaxy and laterality defects becoming more common, and among individuals with Trisomy 18, nearly all will have CHD, usually in the form of septal defects. Approximately one-third of females with Turner syndrome, or monosomy X, have CHD. The malformations are usually of the left-sided cardiac structures, and the most common diagnoses include bicuspid aortic valve, aortic stenosis, hypoplastic left heart syndrome, and coarctation of the aorta. In males with Klinefelter syndrome, or 47, XXY, there is a fifty percent incidence of CHD, with patent ductus arteriosus and atrial septal defects prevailing [5]. These and other less common chromosomal defects are detected in patients with CHD since the advent of the chromosomal G-banded karyotype. However, conventional karyotype analysis has a resolution of only five to ten megabases, limiting this technique in its ability to detect smaller chromosomal anomalies.

With the development of fluorescence in situ hybridization (FISH), a technique in which fluorescent labeled probes are hybridized to metaphase chromosomes to detect small submicroscopic chromosomal deletions and duplications, several syndromes caused by chromosomal abnormalities have been elucidated (Table **1**). Two classic examples include the 22q11 deletion syndrome and Williams-Beuren syndrome. The 22q11 deletion syndrome (also known as DiGeorge, velo-cardio-facial and conotruncal anomaly face syndromes) is caused by a microscopic deletion on chromosome 22q11.2, and leads to cardiac malformations along with thymic and parathyroid hypoplasia and characteristic dysmorphic facies, due to abnormal pharyngeal arch development [6]. The most common cardiac malformations are interrupted aortic arch, truncus arteriosus, and tetralogy of Fallot, and routine genetic testing is now performed in patients with these heart lesions [7]. Williams-Beuren syndrome, characterized by cardiac defects, most commonly supravalvar aortic and pulmonary stenosis as well as peripheral pulmonary stenosis, in addition to typical elfin facies, infantile hypercalcemia, renal involvement, and cognitive disability, is due to microdeletion of chromosome 7p11.23 and is also detectable by FISH [8]. The cardio-vascular defects in this syndrome were found to be due to loss of elastin, and mutations in elastin were identified in individuals with isolated supravalvar aortic stenosis [9, 10]. Although FISH is a powerful technique, it can only be utilized when there is a clinical suspicion that the constellation of symptoms is caused by a specific microdeletion, allowing one to target this area of interest. As such, FISH cannot be applied genome-wide to find novel chromosomal abnormalities.

## SINGLE GENE MUTATIONS

### Single Gene Defects Associated with Syndromes

With advances in genetic technology and the completion of the Human Genome Project, single gene defects leading to syndromes associated with congenital heart disease have been elucidated and they are summarized in Table **2**. Some of the earliest work was the discovery that mutation of *Fibrillin 1* (FBN1) was the cause of Marfan syndrome, which is characterized by progressive aortic root dilation with a predisposition to dissection, lens dislocation, and skeletal anomalies [11]. The genetic etiology was discovered using traditional positional cloning approaches and required the identification of a large kindred with multiple affected members along with a unique phenotype. Since then the genetic basis of numerous syndromes have been identified and each is characterized by a unique constellation of birth defects. Holt-Oram syndrome, characterized by atrial and ventricular septal defects, progressive atrioventricular conduction system disease, and radial limb and thumb anomalies, is associated with mutations in the transcription factor, TBX5 [12]. Alagille syndrome, caused by mutations in *JAG1*, a gene encoding a ligand in the Notch signaling pathway, is characterized by intrahepatic bile duct paucity and cardiovascular malformations, including peripheral pulmonic stenosis, pulmonary valve stenosis, and tetralogy of Fallot [13, 14]. Consistent with this, mutations in a NOTCH receptor, NOTCH2, have also been identified in subjects with Alagille syndrome [15]. The phenotype of Noonan syndrome consists of cardiac defects, typically pulmonary valve stenosis and hypertrophic cardiomyopathy, as well as cognitive disability, characteristic facies, and bleeding disorders. Initially, mutations in *PTPN11*, a gene involved in Ras signaling, were identified to be the cause of 50% of cases [16]. Subsequent studies have found that mutations of other genes involved in the Ras signaling pathway including RAF1, SOS1, and KRAS were also associated with a similar spectrum of disease [17-20]. In addition, LEOPARD and Costello syndromes, which exhibit a similar phenotype as Noonan syndrome, are the result of mutations in Ras signaling pathway members [21-24]. Another syndrome characterized by dysmorphic facies and digit anomalies along with congenital heart disease (specifically patent ductus arteriosus) was found to be caused by mutation in the transcription factor, TFAP2β using traditional approaches after the identification of large kindreds [25]. Lastly, heterotaxy syndrome, which is randomization of cardiac, pulmonary and gastrointestinal situs, is frequently associated with congenital heart disease, specifically atrioventricular septal defects, and transposed great arteries. A subset of these cases have been identified to be caused by mutations in ZIC3, CFC1, ACVR2B, and LEFTYA, genes that regulate left-right asymmetry in the developing embryo [26].

### Single Gene Defects Associated with Non-syndromic Cardiac Malformations

More recently, single gene defects associated with isolated or non-syndromic congenital heart disease have been discovered (Table **3**). Kindred studies revealed that mutations in *NKX2.5* lead to isolated atrial septal defects with atrioventricular conduction delay [27]. Mutations in *GATA4*, a zinc finger transcription factor known to interact with *NKX2.5*, have been linked to isolated atrial septal defects without conduction system abnormalities. Interestingly, a mutation in Gata4 specifically disrupted an interaction with *TBX5* suggesting that mutations in any of these interacting transcription factors can lead to CHD [28]. Myosin heavy chain 6 (*MYH6) *mutations have been identified as another cause of atrial septal defects [29]. MYH6 is known to be activated by TBX5 and GATA4, suggesting that mutations in the common downstream targets of these genes may be a cause of cardiac septation defects. Several genes have been implicated in the genetic etiology of atrioventricular septation defects including CRELD1, ALK2, and BMPR2 [30-32]. Additionally, mutations in *NOTCH1* have been identified as a cause of aortic valve malformations, including bicuspid aortic valve and early aortic valve calcification, via genome-wide linkage analysis of an affected family. Interestingly, family members with trileaflet aortic valves and NOTCH1 mutations also developed early valve calcification, indicating that NOTCH1 also plays a role in valvular calcification [33]. The identification of more severe cyanotic forms of non-syndromic CHD has been limited. The gene PROSIT240 was found to be disrupted by a balanced translocation in a patient with d-transposition of the great vessels and mental retardation, and subsequently additional patients with isolated d-TGA were found to have mutations in this gene [34]. These new developments demonstrate that single-gene defects can lead to isolated congenital heart disease, and reveal more about molecular pathways important in cardiac morphogenesis.

## COPY NUMBER VARIATIONS IN CONGENITAL HEART DISEASE

Despite these advances and discoveries, the vast majority of individuals with CHD do not have single gene defects. With the sequencing of the human genome, new information about genetic diversity has been revealed. One type of genetic variation, the copy number variation (CNV), consists of intermediate-size duplications and deletions that lead to changes in gene dosage and affect about 12% of the human genome [35]. CNV are considered polymorphisms when present in >1% of the population, and are more likely to be disease-associated when occurring in <1% of individuals. Comparative genomic hybridization (CGH), a DNA microarray-based methodology can detect these submicroscopic CNV genome-wide. 

### Copy Number Variation in Human Disease

The presence of CNV encompassing a variety of different genomic regions has been shown to be strongly associated with diseases such as autism, schizophrenia, and developmental delay, along with being identified in individuals with multiple anomalies. Sebat and colleagues performed whole genome array CGH on subjects with autism spectrum disorders and their unaffected siblings and found that *de novo* but individually rare CNVs were significantly associated with autism [36]. More recently, a microdeletion or reciprocal microduplication of chromosome 16p11.2 was identified by array CGH in a large population of families to be significantly associated with autism in 1% of cases [37]. Similar results have been obtained using array CGH in individuals with schizophrenia where rare microdeletions and microduplications are present significantly more often in individuals with schizophrenia than in related controls. These CNV predominantly modified genes involved in neurodevelopment, suggesting that they are contributing to the development of schizophrenia [38, 39]. Array CGH has also been used to study idiopathic mental retardation, as well as mental retardation associated with dysmorphism. Multiple studies have detected abnormal chromosomal copy number associated with these clinical phenotypes, ranging from 4% to 17% of those studied [40, 41]. Across a wide spectrum of diseases, array CGH has developed into a useful tool to detect submicroscopic chromosomal anomalies that lead to disease or increase disease susceptibility. 

### Copy Number Variation in Congenital Heart Disease

Unique copy number variations are associated with congenital cardiac malformations. Initial studies have identified CNVs in children with CHD along with multiple other congenital anomalies. CHARGE syndrome, a constellation of anomalies including coloboma of the eye, heart defects including conotruncal and aortic arch malformations, choanal atresia, growth retardation, and ear abnormalities, was recently found to be due to a microdeletion on chromosome 8p21 [42]. Whole-genome array was initially used to screen the entire genome of individuals with CHARGE syndrome and detected a 2.3 Mb microdeletion on chromosome 8p21 in one individual. Subsequent sequencing of the critical region revealed heterozygous mutations in *CHD7*, a gene which encodes for a chromodomain helicase DNA-binding protein that is expressed during embryogenesis, in 10 subjects, suggesting that *CHD7* haploinsufficiency leads to CHARGE syndrome [42]. Subsequent studies, identified pathogenic mutations in *CHD7* in over 50% of a population of 110 individuals with CHARGE syndrome (Table **2**) [43]. Array CGH helped to identify the gene responsible for the majority of cases of this syndrome, demonstrating the importance of this methodology in gene discovery, especially when studying rare diseases.

Using array CGH, Thienpont and colleagues studied sixty patients with CHD and other birth anomalies and normal chromosomes by standard cytogenetics and detected rare CNV in 30%. While some of these CNV were in regions known to contain genes critical for cardiac development such as *NKX2.5* and *NOTCH1*, the majority occurred where no known cardiac genes were located [44]. Causation was supported when CNV contained genes important in cardiac development or when they arose *de novo*, and this analysis suggested that 17% of the patient population harbored causative CNV. The remaining 13% of CNVs were inherited from unaffected parents, so causation could not be established. This study demonstrated that CNV are associated with CHD that occurs in association with other anomalies or developmental delay.

In another study, high-resolution whole-genome array CGH was performed to detect rare CNV in 40 individuals with CHD and normal karyotypes. Twenty had CHD and other anomalies or developmental delay, while the others had isolated CHD. Large causative CNV were identified in five individuals (25%), all with CHD and neurological abnormalities or developmental delay. The risk of having disease-causing CNV increased to 45% in those with neurological abnormalities or developmental delay. These results demonstrate that large and likely causative CNV exist in individuals with CHD especially when developmental delay or neurological anomalies are also present [45]. These recent studies demonstrate that using whole-genome array CGH to detect disease-associated CNV in individuals with CHD and neurologic anomalies can be useful if conventional genetic evaluation including karyotype is normal.

Copy number variations have also been identified in individuals with isolated CHD. Erdogan and colleagues studied 105 individuals with various types of isolated CHD and no other anomalies or evidence of developmental delay to whole genome array CGH and detected 18 rare CNV. The majority were duplications, in contrast to those found in syndromic CHD, which are predominantly deletions. Additionally, 44% were familial, also occurring in parents with no evidence of CHD, perhaps indicating that these CNV increase susceptibility to CHD but require other modifying factors to manifest the phenotype [46]. This demonstrates that rare CNV may be an important genetic contributor to isolated CHD, but in light of the significant cost for array CGH and inconclusive results to provide accurate genetic counseling, it is not yet clinically indicated in cases of isolated CHD.

## SINGLE NUCLEOTIDE POLYMORPHISMS IN CONGENITAL HEART DISEASE

While copy number variations represent one type of genetic diversity, single nucleotide polymorphisms (SNPs) represent another. SNPs are changes in single nucleotides found throughout the genome; data from the Human Genome Project estimates that 90% of human genetic variation results from SNPs [47]. These may occur in coding or non-coding regions of the genome, and each SNP does not necessarily affect gene function. A collection of SNPs in a particular genomic region each of which is highly non-randomly associated with the others is referred to as a haplotype. Some SNPs have been shown to modify response to pharmaceutical agents, while others have been implicated in susceptibility to disease. Whole genome association studies have detected SNPs that increase susceptibility to common diseases such as cancer, diabetes, and coronary artery disease, and replication studies have confirmed these associations in multiple cohorts. SNPs that influence the development of CHD have also been identified, though the field is still in the early stages of development. The number of subjects with CHD that can be studied is relatively small due to its relative infrequency, significant phenotypic variability, and the early lethality that existed only until the last couple of decades, as such the statistical power that can be generated from whole genome studies is low. As such until now, SNP association studies in CHD have focused on detecting polymorphisms in candidate genes thought to be involved in the development of CHD.

The role of folate metabolism in the development of CHD is one area where initial association studies focused. Maternal folate supplementation is known to reduce the incidence of neural tube defects in offspring, and by a similar mechanism, appears to decrease the incidence of CHD [48]. A common polymorphism in the methylenetetrahydrofolate reductase (MTHFR) gene at position 677 changes a cytosine to a thymine (C677T) and leads to decreased activity of the enzyme. This SNP, found in about 10% of individuals of Northern European origin, has been investigated in multiple studies to determine if it confers an increased risk for CHD. A recent meta-analysis examining all studies of this MTHFR polymorphism failed to find an association between the presence of the homozygous C677T allele in either mothers or offspring and increased risk for CHD [49]. Due to the small sample sizes in the individual studies, heterogeneity in types of CHD, and unknown presence of maternal folic acid supplementation, these results remain inconclusive, and further study is required.

In numerous mouse models, vascular endothelial growth factor (VEGF) has been shown to be critical for normal heart development. Three SNPs in the promoter of *VEGF* (C2578A, G1154A, and C634G) had previously been shown to be a modifier of 22q11 deletion syndrome in a candidate-gene based study and this haplotype is associated with lower VEGF levels *in vivo* [50, 51]. Following this, family based studies using 148 trios found that the low expression VEGF haplotype was transmitted more often than expected to affected children suggesting that it may play a role in the development of isolated TOF [52]. This has been followed by the examination of VEGF SNPs with other types of CHD. The polymorphism at position -634 was found to be associated with ventricular septal defects in a Chinese population [53] and two other VEGF SNPs (-460T/C and +405G/C) also associated with decreased VEGF expression have been reported to be associated with valvuloseptal defects in a small case-control study [54]. Similar to as was seen with polymorphisms in MTHFR, a recent meta-analysis failed to demonstrate an association with VEGF genetic variations and CHD [55]. 

## FUTURE DIRECTIONS

From congenital heart defects due to chromosomal aneuploidy to single gene defects causing isolated CHD, genetics has a much greater influence on the development of various types of CHD than previously appreciated. We are just beginning to understand the significance of copy number variations in CHD, and this field will likely serve as an important mechanism for gene discovery as the resolution of array CGH improves. Although whole-genome association studies using SNPs have been useful in studies of coronary artery disease, the relatively uncommon incidence of CHD and its phenotypic heterogeneity may limit their usefulness in single institution studies due to lack of power. Candidate gene association studies for etiologic and susceptibility genes in CHD will continue to reveal new genetic variations that may modify risk, but these studies will require replication in ethnically diverse populations to be validated. As the cost of DNA sequencing decreases, we will ultimately be able to sequence the entire genome, which may lead to an increase rate of gene discovery in CHD. As new genetic abnormalities associated with CHD are identified, development of international collaborative databases with phenotypic information will allow for the study of clinical outcomes and prognosis based on genetic backgrounds. As more knowledge is gained about the influence of genetics on CHD, strategies for the prevention of CHD or prediction of outcome will develop that will prove beneficial for future generations of children affected by CHD.

## Figures and Tables

**Table 1.** Common Syndromes Resulting from Anueploidy and Microdeletions

Syndrome	Cardiac Anomalies	% with CHD	Other Clinical Features
Trisomy 13	ASD, VSD, PDA, HLHS	80%	Microcephaly, holoprosencephaly, scalp defects, severe mental retardation, polydactyly, cleft lip or palate, genitourinary abnormalities, omphalocele, microphthalmia
Trisomy 18	ASD, VSD, PDA, TOF, DORV, CoA, BAV	90-100%	Polyhydramnios, rocker-bottom feet, hypertonia, biliary atresia, severe mental retardation, diaphragmatic hernia, omphalocele
Trisomy 21 (Down syndrome)	ASD, VSD, AVSD, TOF	40-50%	Hypotonia, developmental delay, palmar crease, epicanthal folds
Monosomy X (Turner Syndrome)	CoA, BAV, AS, HLHS	25-35%	Short stature, shield chest with widely spaced nipples, webbed neck, lymphedema, primary amenorrhea
47, XXY (Klinefelter Syndrome)	PDA, ASD, mitral valve prolapse	50%	Tall stature, hypoplastic testes, delayed puberty, variable developmental delay
22q11.2 deletion (DiGeorge Syndrome)	IAA Type B, aortic arch anomalies, truncus arteriosus, TOF	75%	Thymic and parathyroid hypoplasia, immunodeficiency, low-set ears, hypocalcemia, speech and learning disorders, renal anomalies
7q11.23 deletion (Williams-Beuren Syndrome)	Supravalvar AS, PPS	50-85%	Infantile hypercalcemia, elfin facies, social personality, developmental delay, joint contractures, hearing loss

ASD, atrial septal defect; VSD, ventricular septal defect; PDA, patent ductus arteriosus; HLHS, hypoplastic left heart syndrome; TOF, tetralogy of Fallot; DORV, double outlet right ventricle; CoA, coarctation of aorta; BAV, bicuspid aortic valve; AVSD, atrioventricular septal defect; IAA, interrupted aortic arch; AS, aortic stenosis; PPS, peripheral pulmonic stenosis.

**Table 2.** Common Syndromes Associated with CHD Resulting from Single Gene Defects

Syndrome	Cardiac Anomalies	Other Clinical Features	Causative Gene(s)
Noonan Syndrome	PS with dysplastic pulmonary valve, AVSD, HCM, CoA	Short stature, webbed neck, shield chest, developmental delay, cryptorchidism, abnormal facies	PTPN11, KRAS, RAF1, SOS1
Costello Syndrome	PS, HCM, cardiac conduction abnormalities	Short stature, developmental delay, coarse facies, nasolabial papillomata, increased risk of solid organ carcinoma	HRAS
LEOPARD Syndrome	PS and cardiac conduction abnormalities	Lentigines, hypertelorism, abnormal genitalia, growth retardation, sensorineural deafness	PTPN11, RAF1
Alagille Syndrome	PS, TOF, ASD, peripheral pulmonary stenosis	Bile duct paucity, cholestasis, typical facies, butterfly vertebrae, ocular anomalies, growth delay, hearing loss, horseshoe kidney	JAG1, NOTCH2
Marfan Syndrome	Aortic root dilatation and dissection, mitral valve prolapse	Tall stature, arachnodactyly, pectus abnormality, scoliosis, ectopia lentis, spontaneous pneumothorax, striae, dural ectasia	FBLN, TGFBR1, TGFBR2
Holt-Oram Syndrome	ASD, VSD, AVSD, progressive AV conduction system disease	Preaxial radial ray malformations (thumb abnormalities, radial dysplasia)	TBX5
Heterotaxy Syndrome	DILV, DORV, d-TGA, AVSD	intestinal malrotation	ZIC3, CFC1
Char Syndrome	PDA	Dysmorphic facies and digit anomalies	TFAP2b
CHARGE Syndrome	ASD, VSD, valve defects	Coloboma, choanal atresia, developmental delay, genital and/or urinary anomalies	CHD7, SEMA3E

PS, pulmonic valve stenosis; AVSD, atrioventricular septal defect; HCM, hypertrophic cardiomyopathy; CoA, coarctation of aorta; TOF, tetralogy of Fallot; ASD, atrial septal defect; VSD, ventricular septal defect; AV, atrioventricular; DILV, double inlet left ventricle; DORV, double outlet right ventricle; TGA, transposition of the great arteries; PDA, patent ductus arteriosus.

**Table 3.** Non-Syndromic CHD Resulting from Single Gene Defects

Cardiac Anomalies	Gene
ASD, atrioventricular conduction delay, TOF, tricuspid valve abnormalities	NKX2.5
ASD, VSD	GATA4
ASD, hypertrophic cardiomyopathy	MYH6
Cardiac septation defects associated with PHTN	BMPR2
Endocardial cushion defects	CRELD1, ALK2
BAV, early valve calcification	NOTCH1
d-TGA	PROSIT-240

ASD, atrial septal defect; TOF, tetralogy of Fallot; VSD, ventricular septal defect; TGA, transposition of the great arteries; BAV, bicuspid aortic valve; PHTN, pulmonary hypertension
